# Evaluation of in vitro susceptibility to sparteine in four strains of *Mycobacterium tuberculosis*

**DOI:** 10.17843/rpmesp.2022.391.10136

**Published:** 2022-03-31

**Authors:** Manuel Hidalgo, Percy Asmat Marrufo, Pedro Lezama Asencio, Cynthia Ramos, Carlos Alfonso Chimoy Tuñoque, Gastón Zolla

**Affiliations:** 1 Universidad Privada Antenor Orrego, La Libertad, Trujillo, Peru. Universidad Privada Antenor Orrego Universidad Privada Antenor Orrego La Libertad Trujillo Peru; 2 Laboratorio de Referencia Regional La Libertad, Peru. Laboratorio de Referencia Regional La Libertad Peru; 3 Laboratorio de Fisiología Vegetal de la Universidad Nacional Agraria La Molina, Lima, Peru. Laboratorio de Fisiología Vegetal de la Universidad Nacional Agraria La Molina Lima Peru

**Keywords:** *Mycobacterium tuberculosis*, Tuberculosis, Resistant Tuberculosis, Sparteine, Sparteine Sulfate, Alkaloid

## Abstract

Sparteine is an alkaloid with bacteriostatic activity on the genus Mycobacterium. The aim of this study was to evaluate the antimicrobial activity of sparteine on the growth of 4 ATCC strains of Mycobacterium tuberculosis (susceptible, resistant to isoniazid, resistant to rifampicin and multidrug-resistant) *in vitro*. Validation of bactericidal activity of sparteine sulfate was carried out through an adaptation of the Microscopic-Observation Drug-Susceptibility (MODS) method according to the guidelines of the Peruvian National Health Institute. The results demonstrate that at concentrations of 25; 50 and 100 Mm of sparteine sulfate, there is no development of colony-forming units in any of the 4 evaluated strains. Our results demonstrate the potential in vitro antimicrobial effect of sparteine on multidrug-resistant tuberculosis.

## INTRODUCTION

Tuberculosis (TB) is caused by *Mycobacterium tuberculosis* and affects one-third of the world’s population; approximately 10 million people were infected and 1.4 million died in 2019 ^1^. Treatment is usually insufficient due to the emergence of multidrug-resistant TB (MDR-TB) and extensively drug-resistant TB (XDR-TB) cases, which is why this disease is considered a major global health problem ^2^. According to the World Tuberculosis Report ^1^, Peru is the country most affected by MDR-TB in Latin America, with 30% of the cases, which have been increasing in recent years and include a high incidence of XDR-TB cases in more than half of the regions throughout the country. This has led to TB being considered a health priority, and for this reason it is diagnosed and treated free of charge by the Peruvian state health institutions ^3^.

The emergence of multiple drug resistance pathways has prompted the study and search for new compounds for the effective treatment of TB in order to control drug resistance ^4^. Different natural products have been discovered that possess activity against different resistant strains of *M. tuberculosis*, among which alkaloids stand out ^5^. Ambiguine, derived from *Fischerella ambigua*, significantly inhibits *M. tuberculosis* H37RV with a minimum inhibitory concentration (MIC) of 6.6 µM ^6^. On the other hand, cryptolepine, an indoloquinoline alkaloid extracted from *Cryptolepis sanguinolenta* have shown activity against *M. fortuitum*, with an MIC of 16 ug/mL ^7^. Other alkaloids, including indoles, pyrroles, carbazoles, indoloquinolines, manzamines, quinolines and isoquinolines, have antimicrobial effect on different species of mycobacteria ^5^. Among these compounds are the quinolizidine alkaloids, which have demonstrated anti-TB activity ^8^. Sparteine, in particular, has shown bacteriostatic activity on *Mycobacterium phlei* ^9^, a bacterium with high phylogenetic similarity to *M. tuberculosis*, as revealed by 23S ribosomal RNA analysis, with an identity of 90-100% and an expected BLAST value (E value) = 0 ^10^.

Given the antimicrobial activity of sparteine on *M. phlei*, and after *in vivo* and clinical trials, this alkaloid, or its derivatives, could be used against *M. tuberculosis*. Therefore, this study aimed to evaluate the antimicrobial *in vitro* effect of sparteine on the growth of four ATCC strains of *Mycobacterium tuberculosis* with different degrees of antibiotic resistance, using culture and susceptibility to antituberculosis drugs by microscopic observation (MODS) assay.

**KEY MESSAGES**

Motivation for the study: Peru is the country with the highest incidence of multidrug-resistant tuberculosis in Latin America. This disease can be treated using quinolizidine alkaloids, which have antimicrobial activity in *Mycobacterium phlei*.

Main findings: This study demonstrates that sparteine has *in vitro* antimicrobial effect on ATCC strains of resistant and multidrug-resistant *Mycobacterium tuberculosis*.

Implications: Sparteine shows *in vitro* effect against *M. tuberculosis*; therefore, in vivo assays and clinical studies are needed to demonstrate its efficacy in the treatment of tuberculosis.

## THE STUDY

### Biological material

To evaluate the activity of sparteine sulfate, young cultures of 28 days of incubation were grown in Lowenstein-Jensen medium at the Mycobacteria Laboratory of the Regional Referral Laboratory of La Libertad (LRRLL). The strains used were:

*Mycobacterium tuberculosis* strain sensitive to isoniazid and rifampicin H37Rv (ATCC 27294).

*Mycobacterium tuberculosis* strain resistant to isoniazid (ATCC 35822).

*Mycobacterium tuberculosis* strain resistant to rifampicin (ATCC 35838).

*Mycobacterium tuberculosis* MDR strain (ATCC 35821).

### Preparation of the culture medium and the solutions with sparteine sulfate

**Figure 1:**
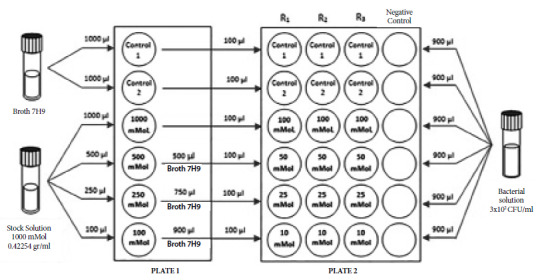
Flow chart of the evaluation of the bactericidal activity of sparteine sulfate against *M. tuberculosis* by microscopic observation (MODS). Middlebrook 7H9 base broth enriched with OADC (Oleic Albumin Dextrose Catalase) was prepared. The base broth was used to prepare the controls. Sparteine concentrations were obtained from dilution of a 1000 mMol stock solution to produce concentrations of 1; 2.5; 5; 10; 25; 50 and 100 mMol. The bacterial suspension was added to the culture media by adjusting the turbidity to Mc Farland scale 1 (3 x 10^8^ CFU/mL).

The evaluation of the bactericidal activity of sparteine sulfate (SS) was carried out using an adaptation of the culture and susceptibility to antituberculosis drugs assay method by MODS, according to the procedure described in the technical guideline prepared by the Instituto Nacional de Salud ^11^. This guideline is based on the observation of the characteristic cords of *M. tuberculosis* when it grows in liquid medium, by means of an inverted light microscope. The flow chart of the SS bactericidal activity evaluation by microscopic observation is described in Figure 1.

Middlebrook 7H9 broth enriched with 10% OADC (Oleic Albumin Dextrose Catalase) was used as the base for the culture medium. Based on the concentrations used in the Wink study ^9^, SS was added to the medium in microplates, to obtain an intermediate concentration of SS equivalent to: 1000; 500; 250; 100; 50; 25 and 10 milliMol. Finally, in another microplate, we placed an aliquot of 100 µL of each previously prepared concentration. Each strain was evaluated in a microplate, in which an aliquot was placed in triplicate (Figure 1). The microplates were placed inside a Ziplock®-type transparent polyethylene bag and protected from light until further evaluation according to the MODS protocol ^11^.

### Preparation of *M. tuberculosis* bacterial suspension

*M. tuberculosis* colonies were collected in tubes with 7H9 broth using an inoculation loop. The suspensions were homogenized by vortexing, and after 30 min of rest, the supernatant was transferred to a tube with 7H9 broth adjusting the turbidity to Mc Farland scale 1 equivalent to 3 x 10^8^ CFU/mL.

### Evaluation of the bactericidal activity of sparteine sulfate against *M. tuberculosis*.

First, 5 mL of Middlebrook 7H9 broth base enriched with 10% OADC solution was placed in a screw-capped glass tube. These tubes were inoculated with 5 µL of the *M. tuberculosis* bacterial suspension previously standardized to Mc Farland’s No. 1, after which it was homogenized. The microplates were inoculated with 100 µL of the different SS concentrations, 900µL of the bacterial suspension was placed in the two growth control wells, and in the wells with 100uL of the concentrations to be evaluated. The microplates were placed in a hermetically sealed bag and incubated at 37°C, then they were checked for contamination and presence of *M. tuberculosis* growth ^11^.

### Microplate reading

At 21 days, we inspected the microplates using the inverted light microscope. We first examined the growth control wells and found colony forming units (CFU), which validates the procedure and the positivity of the culture. Then the wells with the various concentrations of SS were inspected. A positive result was considered as the growth of a CFU in the two wells with 10% OADC AL enriched culture medium (growth control). The interpretation of results from microscopic observation is detailed in Table 1. Only plates that showed growth inhibition in all three replicates were reported as sensitive.

**Table 1:** Interpretation of results by culture assay method and susceptibility to antituberculosis drugs by microscopic observation (MODS).

Growth evaluation	Microscopic observation	Interpretation of findings
Control wells	>1 CFU (both wells)	Positive
	0 CFU	Negative
	1 CFU (one well)	Undetermined
	Bacterial or fungal growth	Contaminated
Wells with concentrations of sparteine sulfate	>1 CFU	Resistant
	0 CFU	Sensitive (growth inhibition)
	Bacterial or fungal growth	Contaminated

CFU: Colony forming units.

### Variables and experimental design

The independent variables were *M. tuberculosis* strains (ATCC 27294; ATCC 35822; ATCC 35838; ATCC 35821) and SS concentrations in the culture broth (1; 2.5; 5; 10; 25; 50 and 100mM). The dependent variable was the presence of *M. tuberculosis* colony forming units.

Each experimental unit was composed of 24-well plates, containing *M. tuberculosis* cultures in the described media, with the concentrations specified in Figure 1. Treatment was assigned to each experimental unit following a completely randomized design.

### Statistical analysis

The chi-square test was used to compare the observed proportions between treatments and SS concentrations (supplementary material).

### Ethical criteria

The project is registered in the Health Research Projects platform (PRISA, https://prisa.ins.gob.pe/), with ID: FD73250D-EFA2-4A82-8EC9-B688DC11D304 and research number: 2104. Strains from the Regional Reference Laboratory of La Libertad were used. Once the study was completed, the material was incinerated according to national regulations. No patients took part in this study; therefore, approval by an institutional ethics committee was not required.

## FINDINGS

Our results show normal growth of the four *M. tuberculosis *strains (ATCC 27294; ATCC 35822; ATCC 35838; ATCC 35821) in the wells without SS, and CFUs could be identified (Table 2). The results detailed below were observed in the three evaluated replicates.

**Table 2:** Results of the evaluation of the bactericidal activity of sparteine sulfate against *M. tuberculosis* by culture assay and susceptibility to antituberculosis drugs by microscopic observation (MODS).

*Mycobacterium tuberculosis* strain	Final sparteine sulfate concentration (mMol)						
	1	2.5	5	10	25	50	100
Control	+	+	+	+	+	+	+
Sensitive strain H37Rv (ATCC 27294)	+	+	+	+	-	-	-
Isoniazid resistant strain (ATCC 35822)	+	+	+	+	-	-	-
Rifampicin resistant strain (ATCC 35838)	+	+	+	+	-	-	-
MDR strain (ATCC 35821)	+	+	+	+	-	-	-

(+) Growth of *M. tuberculosis* showing colony-forming units in all three replicates.

(-) No growth of *M. tuberculosis*, no colony forming units in the three replicates.

mMol: millimolar.

**Figure 2:**
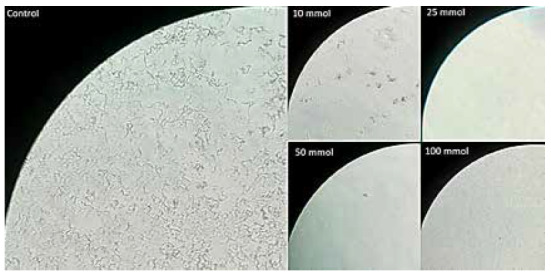
Broth growth of *M. tuberculosis* MDR strain (ATCC 35821) subjected to different concentrations of sparteine sulfate. The control photograph shows the characteristic cords which indicate growth in liquid medium of *M. tuberculosis*. The photos with concentrations of 10, 25, 50 and 100 mmol show inhibition of *M. tuberculosis* growth. The photographs were taken using an Olympus inverted light microscope with 1000x magnification.

Likewise, during microscopic observation, CFU growth was detected in all *M. tuberculosis* strains (ATCC 27294; ATCC 35822; ATCC 35838; ATCC 35821) at concentrations of 1; 2.5; 5 and 10 mM of sparteine sulfate. In contrast, we did not detect the cords that indicate the presence of CFU (supplementary material) when evaluating the growth of the same strains in the wells with SS concentrations of 25; 50 and 100 mM. This lack of growth showed the inhibition of *Mycobacterium tuberculosis* growth by the antimicrobial effect of SS. Microscopic findings are shown in Figure 2.

## DISCUSSION

All the evaluated *M. tuberculosis* strains in the wells with SS concentrations of 1 mM; 2.5 mM; 5 mM and 10 mM showed growth. However, growth was inhibited by SS at concentrations of 25mM, 50mM and 100mM, which have antimicrobial action against the following *M. tuberculosis* strains grown in broth: ATCC 27294, ATCC 35822, ATCC 35838 and ATCC 35821. The acquisition of antimicrobial resistance is a natural biological occurrence, which derives from specific chromosomal mutations and is a different and irreversible process for each drug ^2^. However, the emergence of MDR strains has generated the need to search for new diagnostic and treatment methods ^12^.

These results contrast with those obtained for the quinolizidine alkaloid bis-1-oxaquinolizidine, obtained from *Xestospongia exigua*, evaluated with the Microplate Alamar Blue Assay (MABA) colorimetric micromethod which showed an MIC of 3.4 uM against *M. tuberculosis* H37Rv ^13^. On the other hand, sparteine at 20 mM has achieved 100% growth inhibition against *M. phlei* ^9^. Therefore, its antimicrobial activity against *M. tuberculosis* opens the possibility of using it against other species of the genus *Mycobacterium*. This could be of vital importance, because in humans, the rate of infection by non-tuberculous mycobacteria has increased in recent years, as can be evidenced in the following prevalence rates reported by Mora *et al*.^ (^ ^14^., *M. avium* (52%), *M. abscessus* (34%), *M. chelonae* (18%), *M. fortuitum* (16%) and *M. kansasii* (9.1%).

The MODS method has been validated by several studies to detect the presence of *M. tuberculosis* and its susceptibility to antituberculosis drugs such as rifampicin, isoniazid and others such as pyrazinamide, reaching sensitivity and specificity values higher than 90% in TB and MDR-TB compared to the standard agar plate ratio test (APRT) ^15^. It has been successfully used in the Regional Referral Public Health Laboratory of La Libertad, and is the basis for the development of a tuberculosis telediagnosis system and determination of multidrug resistance ^16^.

The limitations of this study include the need for validation of the results by APRT, the evaluation of toxicity in human cells and the comparison with existing drugs recommended for the treatment of TB, such as isoniazid or rifampicin, which have minimum inhibitory concentrations of 0.25 and 2.0 ug/mL, as reported by Schönfeld *et al*. ^17^. In addition, the mechanism of action of sparteine on *M. tuberculosis* should be further studied, since it is a quinolizidine alkaloid and its action could be related to quinolones, with which they share elements of their chemical structure. The bactericidal activity of these drugs is linked to the inhibition of bacterial DNA replication by blocking the ligase domain of DNA gyrase, causing supercoiling and preventing DNA repair, which is why they can be used in cases of MDR-TB ^18^.

In conclusion, this study reports a possible antimicrobial effect of sparteine sulfate on four ATCC strains of *M. tuberculosis* with different degrees of antibiotic resistance (sensitive, isoniazid-resistant, rifampicin-resistant and MDR), under *in vitro* conditions. On the other hand, more standardized assays are required to determine the *in vivo* MIC, as well as clinical studies to determine its effect against TB.
