# Supplementary material for: Evaluation of in vitro susceptibility to sparteine in four strains of Mycobacterium tuberculosis
Source: Rev Peru Med Exp Salud Publica. 2022 Mar 31;39(1):77–82. doi: 10.17843/rpmesp.2022.391.10136 (PMC11397676; doi:10.17843/rpmesp.2022.391.10136)
Supplement: Supplementary material. — Available in the electronic version of the RPMESP. [file rpmesp-39-01-10136-s001.pdf]

MATERIAL SUPLEMENTARIO

1. Prueba chi cuadrado de bondad de ajuste para conteos observados en variable: Crecimiento

Conteos observados y esperados

Categoría	Observado	Proporción de prueba	Esperado	Contribución a chi cuadrado
0	24	0,125	9	25
100	0	0,125	9	9
50	0	0,125	9	9
25	0	0,125	9	9
10	12	0,125	9	1
5	12	0,125	9	1
2,5	12	0,125	9	1
1	12	0,125	9	1

Prueba de chi cuadrado

N	GL	Chi cuad.	Valor p
72	7	56	0,000

Rev Peru Med Exp Salud Publica
https://doi.org/10.17843/rpmesp.2022.391.10136

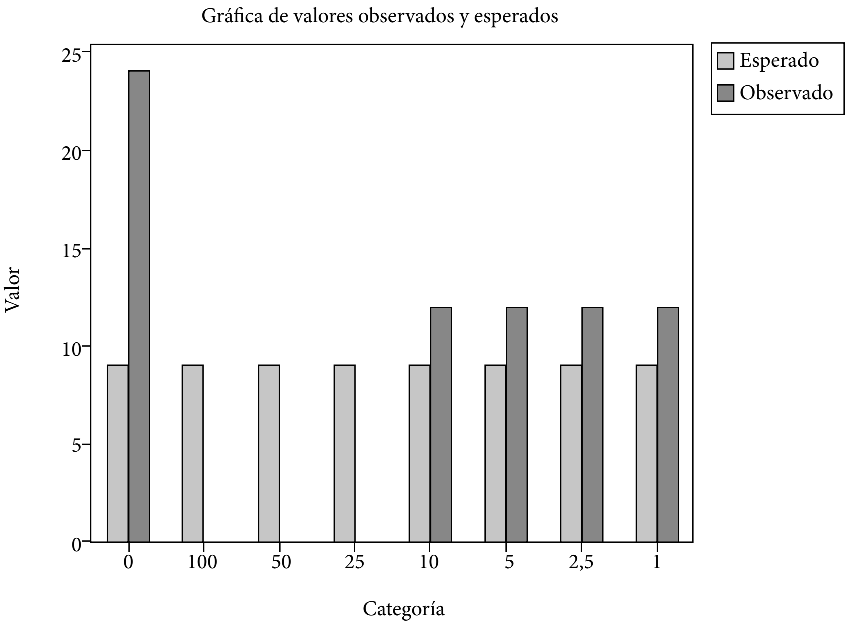

Gráfica de valores observados y esperados.

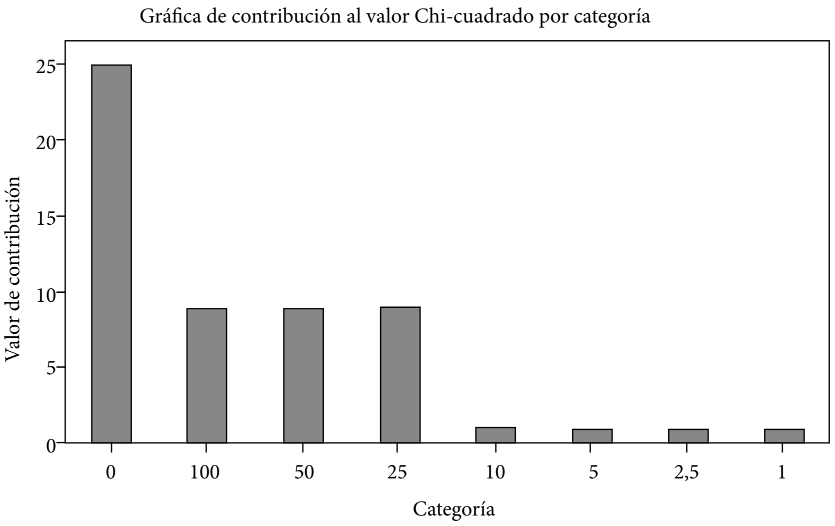

Gráfica de contribución al valor chi cuadrado por categoría

2. Datos en crudo sobre la evaluación del efecto antimicrobiano in vitro de la esparteína contra *Mycobacterium tuberculosis*

Mycobacterium tuberculosis	**Concentración**	**Lectura**		
	**mMol**	**R_1**	**R_2**	**R_3**
Cepa ATCC H37Rv	Control(+) $_1$	+	+	+
	Control(+) $_2$	+	+	+
	100	-	-	-
	50	-	-	-
	25	-	-	-
	10	+	+	+
	5	+	+	+
	2.5	+	+	+
	1	+	+	+
Cepa ATCC 1282	Control(+) $_1$	+	+	+
	Control(+) $_2$	+	+	+
	100	-	-	-
	50	-	-	-
	25	-	-	-
	10	+	+	+
	5	+	+	+
	2.5	+	+	+
	1	+	+	+
Cepa ATCC 3000	Control(+) $_1$	+	+	+
	Control(+) $_2$	+	+	+
	100	-	-	-
	50	-	-	-
	25	-	-	-
	10	+	+	+
	5	+	+	+
	2.5	+	+	+
	1	+	+	+
Cepa ATCC MDR	Control(+) $_1$	+	+	+
	Control(+) $_2$	+	+	+
	100	-	-	-
	50	-	-	-
	25	-	-	-
	10	+	+	+
	5	+	+	+
	2.5	+	+	+
	1	+	+	+
