# Supplementary material for: Evaluation of in vitro susceptibility to sparteine in four strains of Mycobacterium tuberculosis
Source: Rev Peru Med Exp Salud Publica. 2022 Mar 31;39(1):77–82. doi: 10.17843/rpmesp.2022.391.10136 (PMC11397676; doi:10.17843/rpmesp.2022.391.10136)
Supplement: Translation

# Translation: Evaluación de la susceptibilidad *in vitro* a esparteína, en cuatro cepas de *Mycobacterium tuberculosis*

**Authors:** Manuel Hidalgo, Percy Asmat Marrufo, Pedro Lezama Asencio, Cynthia Ramos, Carlos Alfonso Chimoy Tuñoque, Gastón Zolla

**Affiliations:** 1 Universidad Privada Antenor Orrego, La Libertad, Trujillo, Perú.; 2 Laboratorio de Referencia Regional La Libertad, Perú.; 3 Laboratorio de Fisiología Vegetal de la Universidad Nacional Agraria La Molina, Lima, Perú.

**Keywords:** *Mycobacterium tuberculosis*, Tuberculosis, Tuberculosis Resistente, Esparteína, Sulfato de Esparteína, Alcaloide

## Abstract

La esparteína es un alcaloide con actividad bacteriostática sobre el género *Mycobacterium*. El objetivo de este trabajo fue evaluar la acción antimicrobiana de la esparteína en el crecimiento de cuatro cepas ATCC de *Mycobacterium tuberculosis* (susceptible, resistente a isoniazida, resistente a rifampicina y multidrogorresistente) *in vitro*. La evaluación de la actividad bactericida del sulfato de esparteína se realizó a través de una adaptación del método de ensayo de cultivo y susceptibilidad a medicamentos antituberculosos mediante observación microscópica (MODS, por sus siglas en inglés), según el protocolo descrito en el manual técnico elaborado por el Instituto Nacional de Salud. Los resultados demuestran que a concentraciones de 25; 50 y 100 mM de sulfato de esparteína, no se desarrollan unidades formadoras de colonia en las cuatro cepas evaluadas de *Mycobacterium tuberculosis*. Los resultados demuestran el potencial efecto antimicrobiano *in vitro* de la esparteína en la tuberculosis multidrogorresistente.

## INTRODUCCIÓN

La tuberculosis (TB) causada por *Mycobacterium tuberculosis* afecta a un tercio de la población mundial; aproximadamente 10 millones de personas se infectaron en el 2019 y ocasionó la muerte de 1,4 millones de personas ^1^. Dada la aparición de casos de TB multidrogorresistente (TB-MDR) y TB extensivamente resistente (TB-XDR), el tratamiento estándar tiende a ser insuficiente, lo cual convierte a esta enfermedad en un problema mundial de salud importante ^2^. Según el Informe Mundial de Tuberculosis ^1^, en Latinoamérica, Perú es el país más afectado por la TB-MDR, con el 30% de los casos, los cuales se vienen incrementando en los últimos años, e incluyen una alta incidencia de los casos de TB-XDR en más de la mitad de las regiones de todo el país. Lo cual ha determinado que la TB sea considerada como una prioridad sanitaria, y por ello es diagnosticada y tratada de manera gratuita por las instituciones estatales del sistema de salud peruano ^3^.

La aparición de múltiples vías de resistencia a fármacos ha impulsado el estudio y la búsqueda de nuevos compuestos para el tratamiento efectivo de la TB, y así poder controlar la fármacorresistencia ^4^. Se han descubierto diferentes productos naturales que poseen actividad contra diferentes cepas resistentes de *M. tuberculosis*, entre los que destacan los alcaloides ^5^. La ambigüina, derivada de *Fischerella ambigua*, inhibe significativamente a *M. tuberculosis* H37RV con una concentración mínima inhibitoria (CMI) de 6,6 µM ^6^. Por otra parte, la criptolepina, un alcaloide indoloquinolínico extraído de *Cryptolepis sanguinolenta* ha demostrado actividad contra *M. fortuitum*, con una CMI de 16 ug/mL ^7^. Otros alcaloides, incluyendo alcaloides indólicos, pirrólicos, carbazólicos, indoloquinolínicos, manzamínicos, quinolínicos e isoquinolínicos, tienen efecto antimicrobiano en diferentes especies de micobacterias ^5^. Dentro de estos compuestos están los alcaloides quinolizidínicos, que han demostrado actividad anti-TB ^8^. La esparteína, en particular, ha presentado actividad bacteriostática en *Mycobacterium phlei* ^9^, una bacteria con alta similitud filogenética con *M. tuberculosis*, según lo revelan los análisis de ARN ribosomal 23S, una identidad de 90-100% y con valor esperado de BLAST (valor E) = 0 ^10^.

Dada la actividad antimicrobiana de la esparteína en *M. phlei*, y tras los ensayos *in vivo* y clínicos, este alcaloide, o sus derivados, podrían ser utilizados contra *M. tuberculosis*. Por ello, el presente estudio tuvo como objetivo evaluar *in vitro* la acción antimicrobiana de la esparteína sobre el crecimiento de cuatro cepas ATCC de *Mycobacterium tuberculosis* con diferentes grados de resistencia a antibióticos, utilizando el ensayo de cultivo y susceptibilidad a medicamentos antituberculosos mediante observación microscópica (MODS, por sus siglas en ingles).

**MENSAJES CLAVE**

Motivación para realizar el estudio: el Perú es el país con mayor incidencia de tuberculosis multidrogorresistente en Latinoamérica. El tratamiento de esta enfermedad puede realizarse utilizando alcaloides quinolizidínicos, que tienen actividad antimicrobiana en *Mycobacterium phlei.*

Principales hallazgos: este trabajo demuestra que la esparteína tiene efecto antimicrobiano *in vitro* sobre cepas ATCC de *Mycobacterium tuberculosis* resistentes y multidrogorresistentes *in vitro.*

Implicancias: la esparteína muestra efecto *in vitro* contra *M. tuberculosis, *lo que hace necesario la realización de ensayos *in vivo *y estudios clínicos que demuestren su eficacia en el tratamiento de tuberculosis.

## EL ESTUDIO

### Material biológico

Para evaluar la actividad del sulfato de esparteína se emplearon cultivos jóvenes de 28 días de incubación sembrados en medio Lowenstein-Jensen en el Laboratorio de Micobacterias del Laboratorio Referencial Regional de La Libertad (LRRLL). Las cepas empleadas fueron:

Cepa de *Mycobacterium tuberculosis *sensible a isoniacida y rifampicina H37Rv (ATCC 27294).

Cepa de *Mycobacterium tuberculosis* resistente a isoniazida (ATCC 35822).

Cepa de *Mycobacterium tuberculosis *resistente a rifampicina (ATCC 35838).

Cepa de *Mycobacterium tuberculosis *MDR (ATCC 35821).

### Preparación de medio de cultivo y soluciones con sulfato de esparteína

La evaluación de la actividad bactericida del sulfato de esparteína (SE) se realizó a través de una adaptación del método de ensayo de cultivo y susceptibilidad a medicamentos antituberculosos mediante MODS, según el protocolo descrito en el manual técnico elaborado por el Instituto Nacional de Salud ^11^, basado en la observación de los cordones característicos de *M. tuberculosis* cuando crece en medio líquido, mediante el uso de un microscopio luz invertida. El flujograma de la evaluación de la actividad bactericida del SE mediante observación microscópica esta descrito en la Figura 1.

**Figura 1:** Flujograma de la evaluación de la actividad bactericida del sulfato de esparteína contra *M. tuberculosis* mediante observación microscópica (MODS). Se preparó el caldo base Middlebrook 7H9 enriquecido con OADC (Oleic Albumin Dextrose Catalase). El caldo base fue utilizado para preparar los controles. Las concentraciones de esparteína se obtuvieron a partir de la dilución de una solución stock 1000 mMol para generar las concentraciones de 1; 2,5; 5; 10; 25; 50 y 100 mMol. La suspensión bacteriana fue agregada a los medios de cultivo ajustando la turbidez a la escala 1 de Mc Farland (3 x 108 UFC/mL)

La base para el medio de cultivo fue el caldo base Middlebrook 7H9 enriquecido con OADC (Oleic Albumin Dextrose Catalase) al 10%. Tomando como referencia las concentraciones del estudio de Wink ^9^, se añadió SE al medio en microplacas, para obtener una concentración intermedia de SE equivalente a: 1000; 500; 250; 100; 50; 25 y 10 miliMol. Finalmente, en otra microplaca, se colocó una alícuota de 100 µL de cada concentración preparada previamente. Cada cepa se evaluó en una microplaca, en la que se colocó una alícuota por triplicado (Figura 1). Las microplacas fueron puestas en el interior de una bolsa de polietileno transparente con cierre hermético tipo Ziplock**®** y protegidas de la luz hasta su posterior evaluación según el protocolo de MODS ^11^.

### Preparación de la suspensión bacteriana de *M. tuberculosis*

Las colonias de *M. tuberculosis* fueron colectadas en tubos con caldo 7H9 mediante un asa bacteriológica. Las suspensiones fueron homogenizadas en vórtex, y tras 30 min de reposo, el sobrenadante de la suspensión fue transferido a un tubo con caldo 7H9 ajustando la turbidez a la escala 1 de Mc Farland equivalente a 3 x 108 UFC/mL.

### Evaluación de la actividad bactericida de sulfato de esparteína frente a *M. tuberculosis*

Se colocaron 5 mL de caldo base Middlebrook 7H9 enriquecido con solución OADC al 10% en un tubo de vidrio con tapa rosca. En estos tubos se inocularon 5 µL de la suspensión bacteriana de *M. tuberculosis* previamente estandarizada al N.º 1 de Mc Farland, tras lo cual se homogeneizó. Se inocularon las microplacas con los 100 µL de las diferentes concentraciones de SE, con 900µL de la suspensión bacteriana en los dos pozos de control de crecimiento y en los pozos con 100uL de las concentraciones por evaluar. Las microplacas se colocaron en la bolsa con cierre hermético y puestas a incubar a 37 °C, tras lo cual fueron revisadas para evaluar la contaminación y presencia de crecimiento de *M. tuberculosis* ^11^.

### Lectura de las microplacas

A los 21 días, las microplacas fueron observadas con el microscopio de luz invertida. Se revisaron primero los pocillos de control de crecimiento hasta que se detectó la presencia de unidades formadoras de colonias (UFC), lo que indicó la validez del procedimiento y la positividad del cultivo. Luego, se revisaron los pocillos con las diversas concentraciones de SE evaluadas. Un resultado positivo fue tomado como el crecimiento de una UFC en los dos pocillos con medio de cultivo enriquecido con OADC AL 10% (control de crecimiento). La interpretación de resultados de la observación microscópica se detalla en la Tabla 1. Solamente se reportaron como sensibles las placas que mostraron inhibición del crecimiento en las tres repeticiones.

**Tabla 1:** Interpretación de resultados por el método de ensayo de cultivo y susceptibilidad a medicamentos antituberculosos mediante observación microscópica (MODS).

Evaluación de crecimiento	Observación microscópica	Interpretación de los hallazgos
Pocillos control	> 1 UFC (ambos pocillos)	Positivo
	0 UFC	Negativo
	1 UFC (solo un pocillo)	Indeterminado
	Crecimiento de bacterias u hongos	Contaminado
Pocillos con las concentraciones de sulfato de esparteína	> 1 UFC	Resistente
	0 UFC	Sensible (inhibición de crecimiento)
	Crecimiento de bacterias u hongos	Contaminado

UFC: unidad formadora de colonia.

### Variables y diseño experimental

Las variables independientes fueron las cepas de *M. tuberculosis* (ATCC 27294; ATCC 35822; ATCC 35838; ATCC 35821) y las concentraciones de SE en el caldo de cultivo (1; 2,5; 5; 10; 25; 50 y 100mM). La variable dependiente fue la presencia de unidades formadoras de colonias de *M. tuberculosis*.

Cada unidad experimental estuvo compuesta por placas de 24 pozos, conteniendo los cultivos de *M. tuberculosis* en los medios descritos, con las concentraciones especificadas en la Figura 1. Los tratamientos fueron asignados a cada una de las unidades experimentales siguiendo un diseño completamente aleatorio.

### Análisis estadístico

Se utilizó la prueba de prueba de chi cuadrado para comparar las proporciones observadas entre tratamientos y concentraciones de SE (material suplementario).

### Criterios éticos

El proyecto está registrado en la plataforma de Proyectos de Investigación en Salud (PRISA, https://prisa.ins.gob.pe/), con ID: FD73250D-EFA2-4A82-8EC9-B688DC11D304 y número de investigación: 2104. Se utilizó cepas mantenidas en el Laboratorio de Referencia Regional de La Libertad. Una vez concluido el estudio, el material fue incinerado según la normativa nacional. No se utilizaron pacientes en este estudio, por lo cual no se requirió la aprobación de un comité institucional de ética.

## HALLAZGOS

Los resultados demuestran el crecimiento normal de las cuatro cepas de *M. tuberculosis* (ATCC 27294; ATCC 35822; ATCC 35838; ATCC 35821) utilizadas en el estudio en los pocillos sin SE, pudiendo distinguirse la presencia de UFC (Tabla 2). Los resultados detallados a continuación fueron observados en las tres repeticiones evaluadas.

**Tabla 2:** Resultados de la valoración de la actividad bactericida de sulfato de esparteína contra *M. tuberculosis* mediante el ensayo de cultivo y susceptibilidad a medicamentos antituberculosos mediante observación microscópica (MODS).

*Cepa de Mycobacterium tuberculosis*	Concentración final de sulfato de esparteína (mMol)						
	1	2,5	5	10	25	50	100
Control	+	+	+	+	+	+	+
Cepa sensible H37Rv (ATCC 27294)	+	+	+	+	-	-	-
Cepa resistente a isoniazida (ATCC 35822)	+	+	+	+	-	-	-
Cepa resistente a rifampicina (ATCC 35838)	+	+	+	+	-	-	-
Cepa MDR (ATCC 35821)	+	+	+	+	-	-	-

(+) desarrollo de *M. tuberculosis *mostrando unidades formadoras de colonia en las tres repeticiones.

(-) sin desarrollo de* M. tuberculosis, *sin unidades formadoras de colonia en las tres repeticiones.

mMol: milimolar.

Asimismo, en la observación microscópica se pudo detectar el crecimiento de UFC en todas las cepas de *M. tuberculosis* en las concentraciones de 1; 2,5; 5 y 10 mM de sulfato de esparteína en las cepas de *M. tuberculosis* ATCC 27294; ATCC 35822; ATCC 35838; ATCC 35821. En contraste, al evaluar el crecimiento de las mismas cepas en los pocillos sometidos a las concentraciones con 25; 50 y 100 mM de SE, no se detectó la presencia de los cordones que indican la presencia de UFC (material suplementario). Esta ausencia de crecimiento indicó la inhibición de crecimiento de *Mycobacterium tuberculosis* por efecto antimicrobiano del SE. Las observaciones microscópicas son mostradas en la Figura 2.

**Figura 2:** Crecimiento en caldo de la cepa MDR de *M. tuberculosis* (ATCC 35821) sometida a diferentes concentraciones de sulfato de esparteína. La fotografía control muestra el crecimiento de cordones característicos del crecimiento en medio líquido d*e M. tuberculosis*. Las fotos con concentraciones de 10; 25; 50 y 100 mmol, muestran inhibición del crecimiento de *M. tuberculosis.* Las fotografías fueron realizadas utilizando un microscopio de luz invertida Olympus, con 1000 aumentos.

## DISCUSIÓN

Se observó crecimiento de todas las cepas de *M. tuberculosis* evaluadas en los tratamientos de 1 mM; 2,5 mM; 5 mM y 10 mM de SE. Sin embargo, este crecimiento fue inhibido por el SE en concentraciones de 25mM, 50mM y 100mM, las cuales tienen acción antimicrobiana frente a las cepas ATCC 27294, ATCC 35822, ATCC 35838 y ATCC 35821 de *M. tuberculosis* cultivado en caldo. La adquisición de resistencia a los antimicrobianos es una ocurrencia biológica natural, que deriva de mutaciones cromosómicas específicas y son individuales e irreversibles para cada fármaco ^2^. Sin embargo, la aparición de cepas MDR ha generado la necesidad de buscar nuevos métodos de diagnóstico y tratamiento ^12^.

Estos resultados contrastan con los obtenidos para el alcaloide quinolizidínico bis-1-oxaquinolizidina, obtenido de *Xestospongia exigua*, evaluado con el ensayo del micrométodo colorimétrico azul de Alamar (MABA) que mostró una CMI de 3,4 uM contra *M. tuberculosis* H37Rv ^13^. Por otra parte, la esparteína ha alcanzado una inhibición de crecimiento de 100% a 20 mM en *M. phlei* ^(^ ^9^. Por lo cual, su actividad antimicrobiana contra *M. tuberculosis* abre la posibilidad de su uso contra otras especies del género *Mycobacterium*. Esto sería vital, puesto que, en seres humanos, la tasa de infección por micobacterias no tuberculosas se ha incrementado en los últimos años, con prevalencia de infecciones por *M. avium* (52%), *M. abscessus* (34%), *M. chelonae* (18%), *M. fortuitum* (16%) y *M. kansasii* (9,1%), según lo reportado por Mora *et al*. ^14^.

El método MODS ha sido validado por diversos estudios para detectar la presencia y susceptibilidad de *M. tuberculosis* a los fármacos antituberculosos como rifampicina, isoniacida y otros como pirazinamida, alcanzando valores de sensibilidad y especificidad superiores al 90% en TB y MDR-TB en comparación con la prueba estándar del método de proporciones en agar en placa (MPAP) ^15^. Su aplicación ha sido llevada a cabo con éxito en el Laboratorio Regional Referencial de Salud Pública La Libertad siendo la base para el desarrollo de un sistema de telediagnóstico de tuberculosis y determinación de multidrogorresistencia ^16^.

Las limitaciones de este estudio incluyen la necesidad de validación de los resultados mediante MPAP, la evaluación de su toxicidad en células humanas y su comparación con los fármacos existentes de uso recomendado en el tratamiento de TB, como la isoniacida o rifampicina, que tienen concentraciones mínimas inhibitorias de 0,25 y 2,0 ug/mL según reportan Schönfeld *et al*. ^17^. Además, se debe estudiar el mecanismo de acción de la esparteína sobre *M. tuberculosis*, puesto que, al ser parte de los alcaloides quinolizidínicos, su acción podría estar relacionada con las quinolonas, con las cuales comparten elementos de su estructura química. Estos fármacos tienen una actividad bactericida ligada a la inhibición de la replicación del ADN bacteriano al bloquear el dominio ligasa de la ADN girasa, originando superenrollamiento e impidiendo la reparación del ADN, por lo cual se pueden utilizar en los casos de TB-MDR ^18^.

Como conclusión, este estudio muestra un posible efecto antimicrobiano del sulfato de esparteína sobre cuatro cepas ATCC de *M. tuberculosis* con diferentes grados de resistencia a antibióticos (sensible, resistente a isoniacida, resistente a rifampicina y MDR), en condiciones *in vitro*. Por otro lado, se requieren más ensayos estandarizados para determinar la CMI *in vivo*, así como estudios clínicos para determinar su efecto contra la TB.
